# Dissipation and Dietary Risk Assessment of Pydiflumetofen Residues in Soybean

**DOI:** 10.3390/molecules27238465

**Published:** 2022-12-02

**Authors:** Liping Wei, Xingang Hou, Zhiguang Hou, Xiaolong Yu, Xiumei Wang, Qinghui Zhao, Hemin Gao, Hanju Liu, Xiaodong Zheng, Zhongbin Lu

**Affiliations:** 1 College of Plant Protection, Jilin Agricultural University, Changchun 130118, China; 2 College of Science, China Agricultural University, Beijing 100193, China

**Keywords:** pydiflumetofen, soybean, degradation dynamic, dietary risk assessment

## Abstract

In this study, the quick, easy, cheap, effective, rugged, and safe (QuEChERS) method, combined with high-performance liquid chromatography–tandem mass spectrometry, was chosen for detecting pydiflumetofen residues in soybean plants, soybeans and soil, and assessing the risk of short- and long-term dietary intake. Pydiflumetofen concentrations ranging from 0.001–0.5 mg/L exhibited good linearity (*r* > 0.997). At varying doses, the average pydiflumetofen recovery rates and relative standard deviations among soybean plants, soybeans, and soil ranged from 83.9 ± 1.1% to 99.5 ± 3.3% and from 0.77 to 7.77%, respectively. The sensitivity, accuracy, and precision of the chosen methodology met the requirements of pesticide residue analysis. The results of the degradation dynamics test showed that the half-life of pydiflumetofen (t_1/2_) in soybean plants and in soil were 3.6 to 5.7 and from 7.9 to 25.7 d, respectively. Assessment of the concentration of pydiflumetofen residues in soybeans revealed acute and chronic dietary exposure risks of 0.06 and 7.54%, respectively. As these values are very low, pydiflumetofen residues in soybeans present an acceptable risk to public health. The results of this study will help to guide the practical application of pydiflumetofen and minimize the environmental risks associated with its use.

## 1. Introduction

Soybean [*Glycine max* (L) Merr.] is an important cash crop that is widely used as a source of oil, feed, and nutrition; it occupies an important position in global crop production [1]. According to data from China’s National Bureau of Statistics, China’s soybean production and amount of soybean imports in 2020 were 1.96 × 10^7^ t and 1.03 × 10^8^ t, respectively, indicating that more than 80% of soybeans were imported from abroad. Thus, the soybean productivity of China was unable to satisfy the growing needs of its population [2]. During growth soybean is susceptible to insects and to diseases such as root rot and brown spot [3,4], which not only affect the yield, appearance, and quality of the soybean, but also reduce the germination rate and oil content [5,6]. Therefore, the application of pesticides is essential to protect soybeans from pests, diseases, and weeds. However, long-term use of pesticides in large quantities can lead to disease resistance, excessive product residue, and ecological and environmental pollution. In 2012, a study reported that soybean brown spot disease caused by *Cercospora* was found to be resistant to quinone outside inhibitor fungicides such as pyraclostrobin [7].

Pydiflumetofen (Figure S1) is a new type of pyrazole carboxamide fungicide that was developed by Syngenta and belongs to the succinate dehydrogenase inhibitor (SDHI) class. Pydiflumetofen exerts its primary mode of action by inhibiting succinate dehydrogenase, thereby resulting in tricarboxylic acid circulation disorders, which prevent energy metabolism and inhibit the growth of pathogenic bacteria, resulting in their death. Thus, the purpose of disease prevention and control is achieved [8]. As a third-generation SDHI fungicide and a broad-spectrum bactericide, pydiflumetofen shows high efficacy in the prevention and control of nuclear disc bacterial and fungal diseases in soybean, wheat, and a variety of vegetables. Pydiflumetofen has substantial advantages in the control of *Fusarium*, and can be used for the prevention and treatment of soybean brown spot disease, *fusarium* head blight of wheat, rape *sclerotia*, and other diseases [9,10]. Pydiflumetofen was first registered in Argentina in 2017, and is one of the most promising pesticides for SDHI [11]. In 2018, the Joint FAO/WHO Meeting on Pesticide Residues (JMPR) conducted the first toxicological assessment of pydiflumetofen and determined that the acute reference dose (ARfD) of pydiflumetofen was 0.3 mg/kg body weight (bw), and the acceptable daily intake (ADI) value was 0–0.1 mg/kg bw [12].

Pydiflumetofen is used globally to control diseases of fruits, vegetables, and other grains. With the widespread use of pydiflumetofen in crops and its ability to enter the environment and human body through various routes, methods for analyzing residual amounts of pydiflumetofen have attracted much attention. Bian et al. [13] used the quick, easy, cheap, effective, rugged, and safe (QuEChERS) approach combined with high-performance liquid chromatography–tandem mass spectrometry (HPLC–MS/MS) to determine the residual amount of pydiflumetofen in paddy field environments and assessed the dietary risk of pydiflumetofen in rice. Wu et al. [14] used ultra-high performance liquid–chromatography–tandem mass spectrometry (UPLC–MS/MS) to detect the residual amount of pydiflumetofen in wheat ears during the flowering and harvesting stages, and Liu et al. [15] established an analytical method for determining pydiflumetofen residues in seven plant-derived foods (tomatoes, cucumbers, apples, grapes, potatoes, watermelons, and bananas) by combining QuEChERS with UPLC–MS/MS. Zhao et al. [16] used the QuEChERS method with UPLC–MS/MS to detect the residual amount of pydiflumetofen and difenoconazole in banana fruit and assessed the dietary risk thereof. Rong et al. [17] used UPLC–MS/MS to determine pydiflumetofen residues in grapes, potatoes, wheat, milk, pork, and eggs. Kong et al. [18] also used the UPLC–MS/MS method to detect pydiflumetofen in soil samples. In addition, the environmental behavior of pydiflumetofen enantiomers has been reported [19,20]. However, articles have been published exploring the residues and degradation of pydiflumetofen on wheat, watermelon, rice, and banana, which have different growth conditions and climatic conditions in the main growing areas. Therefore, it is necessary to focus on the residues and degradation of pydiflumetofen in soybean. We have recommended MRLs for pydiflumetofen in soybean from the results of the field trials. Although dietary risk assessments for pydiflumetofen in rice and banana have been reported, rice belongs to the category of rice and its products, banana belongs to the category of fruit, and soybean belongs to the category of soybean and its soybean products, which have different daily intakes in the Chinese dietary structure. Therefore, the risk of dietary exposure to pydiflumetofen in soybeans is different. This will help to promote food safety and provide assistance for market supervision.

The purpose of this study was to choose a highly sensitive, reproducible, economical, and practical analytical method for detecting pydiflumetofen residues in soybean plants, soybeans, and soil. The degradation dynamics and residue distribution of pydiflumetofen in soybean plants and soil at three locations were analyzed to assess its stability and persistence in crops and the environment. Moreover, the dietary risk of exposure to pydiflumetofen in soybean was assessed based on field residual data and relevant toxicological parameters. These results will provide guidance for the establishment of a suitable maximum residue limit (MRL) for pydiflumetofen in soybean, as well as parameters for its scientific use and safety in field environments.

## 2. Results and Discussion

### 2.1. Choice of HPLC–MS/MS Conditions

First, the monoisotopic mass of pydiflumetofen was recognized as 425.0271 by checking the relevant literature and calculating it using the Qualitative Analysis Navigator (B.08.00, Agilent Technologies, Santa Clara, CA, USA). Next, to confirm the accuracy of the results, we verified the results obtained above through the method-building process. A 0.1 mg/L standard solution of pydiflumetofen (prepared, in pure acetonitrile) was used to optimized the instrument method settings in HPLC–MS/MS using the C_18_ column, including the ESI mode, precursor ion, fragmentor, product ions, and collision energy, which are key conditions for target compound monitoring. We compared the two modes of ESI+ and ESI− in the 200–500 *m*/*z* range. The results showed that the response of pydiflumetofen was higher in the ESI+ mode than the ESI− mode probably because it was better ionized in the former mode, and the 426.1 *m*/*z* peak was significantly higher than those of other ions. Thus, this was determined as the precursor ion of pydiflumetofen owing to the existence of pydiflumetofen as [M + H] in ESI+. Furthermore, the fragmentor was optimized in selective ion monitoring mode, selecting only the 426.1 *m/z* value. Two characteristic product ions were selected, followed by optimization of these collision energies in the subsequent steps. Finally, pydiflumetofen detection was established using the MRM method.

In pesticide residue detection, acetonitrile: water is the most widely used mobile phase, and the addition of formic acid and ammonium acetate to the aqueous phase can effectively promote ionization and enhance the response of the target compound. Therefore, acetonitrile was selected as the organic phase, and the effects of four aqueous phases, comprising pure water and aqueous solutions of 0.1% formic acid, 0.1% ammonium acetate, and 0.1% formic acid with 5 mmol ammonium acetate on the ionization efficiency of pydiflumetofen, were compared. The results are presented in Figure S2. When acetonitrile: pure water and acetonitrile: 0.1% formic acid solution were the mobile phases, the response value of the pydiflumetofen chromatography peak was low; when acetonitrile: 5.0 mmol ammonium acetate solution was used as the mobile phase, the peak type was not sufficiently sharp, and the retention time was relatively delayed. When acetonitrile: 0.1% formic acid solution with 5.0 mmol ammonium acetate was the mobile phase, the retention time and separation of pydiflumetofen were better, the shape of the chromatographic peak was sharp, and the response value was the highest among those obtained.

### 2.2. Choice of Extraction Conditions

The QuEChERS method has been designed to allow multiresidue analysis, and has been reported to be used for single analytes in other studies. This study required the analysis of different types of extractants and purification agents for the QuEChERS method to obtain satisfactory recoveries. Numerous solvents, such as methanol, acetonitrile, ethyl acetate, and acetone, have been used to extract pesticides from the matrices. The extraction of soybean was compared with and without the addition of water for the four organic solvents. It was found that when methanol and acetonitrile were used as the extraction solution, the solution was relatively turbid, although this was not found after the addition of water. Subsequent extracts of soil were also compared and found to have the same result. Plant substrates contain a large amount of chlorophyll, which causes the color of the extract to become dark; thus, this phenomenon does not occur. The final choice was to add 5 mL water mixed with organic solvent as the extraction solution to facilitate the next step of purification. The result is shown in Figure S3.

**Figure 1:**
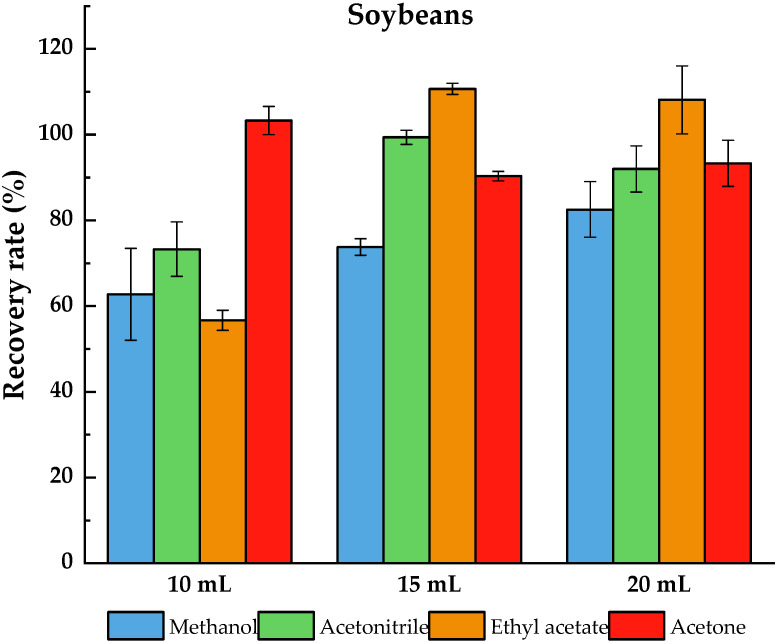

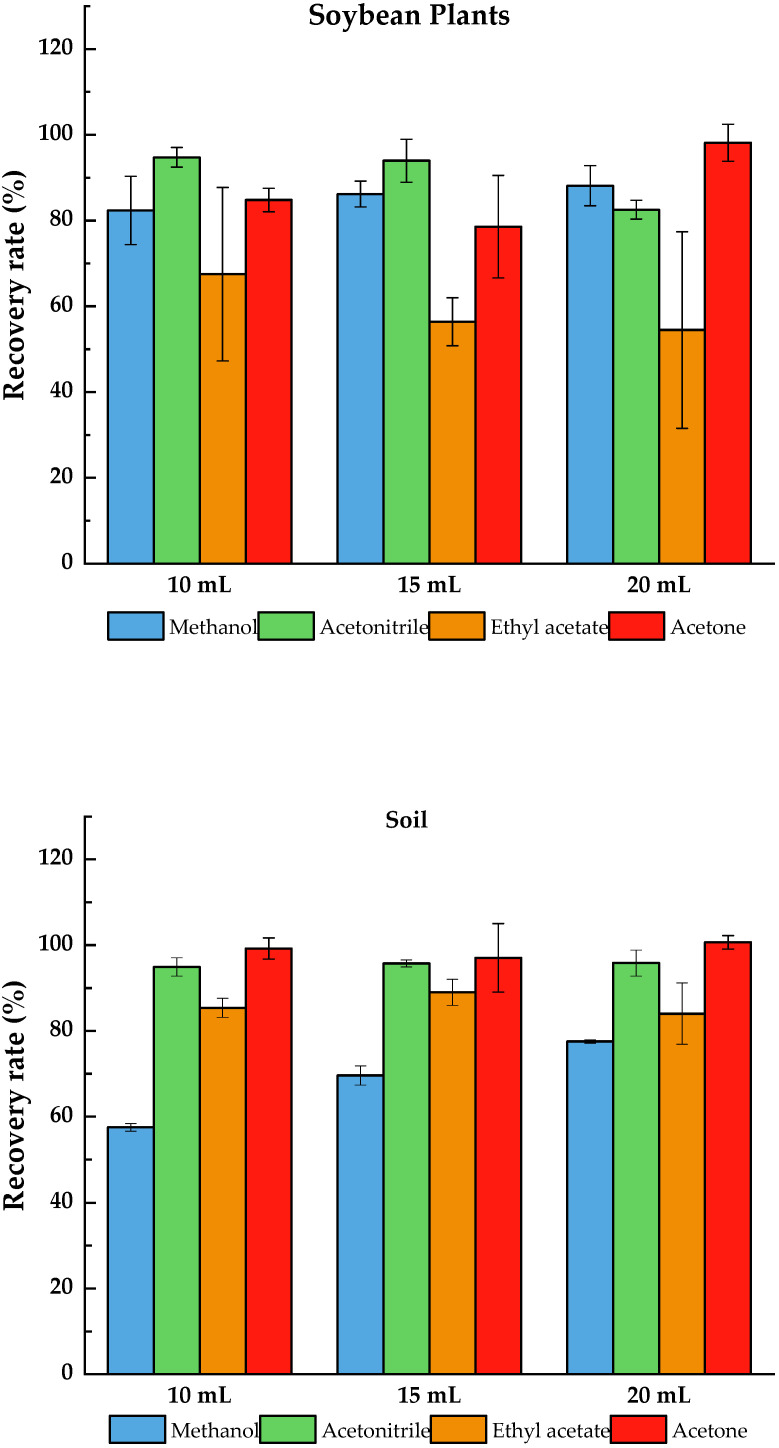
Method efficiency to extract pydiflumetofen from soybeans, soybean plants and soil using different solvents.

Anastassiades et al. [21] showed that when performing pesticide residue extraction, the salinization effect of MgSO_4_ and NaCl with a mass ratio of 4:1 as a salinizing agent is the best; therefore, 4 g MgSO_4_ and 1 g NaCl were selected as the salinizing agents. In this experiment, we compared the effects of aqueous solution of four organic solvents on the extraction efficiency of pydiflumetofen from soybean in three different extraction volumes. The tests results are shown in Figure 1, and the recovery rates were evaluated by the following equation:

(1) $$\mathrm{Recovery}\;\mathrm{rate}\;=\frac{C_{a}}{C_{q}}\times 100$$

where C_a_ was the concentration of the added samples, which was prepared by adding the pydiflumetofen standard to the blank matrix sample, and C_q_ was the concentration of the QC sample, which was prepared using a blank matrix solution.

According to the JMPR report [12], the solubility of pydiflumetofen in methanol is 26 g/L at 25 °C, which is less than other organic solvents; this may be the main reason for the low recovery rate (only 62.7–82.5%). When acetonitrile was used as an extraction agent, the recovery rate was 73.3–99.4%, and when acetone was used as an extraction agent, the recovery rate was 90.3–103.3%. Acetonitrile and acetone exhibited reasonable recovery rates in all three extraction volumes. However, when acetone was used as an extraction agent, the color of the sample solution was darker than that obtained with acetonitrile. This may be because the use of acetone results in higher amounts of co-extracts during the extraction process, which is not conducive to further purification, and causes damage to the life and precision of the instrument. The recovery rate of ethyl acetate as an extraction agent was 108.1–110.7%, and the sample solution was turbid, which may be because ethyl acetate extracts soil and wax in the sample matrix along with the target compound [22]. Furthermore, it is highly volatile and can cause tremendous harm to the tester and environment. In addition, when acetone and ethyl acetate agents were used as extraction agents, the operation steps were cumbersome and solvent conversion was required. Thus, acetonitrile was ultimately selected as the extraction solvent. For extraction volumes of 10, 15, and 20 mL, the recovery rates were 73.3%, 99.4%, and 92.0%, respectively, and the RSDs were 6.4%, 1.6%, and 5.4%, respectively. Thus, 15 mL acetonitrile and 5 mL water were ultimately selected as the extraction agents. 

### 2.3. Choice of Purification Conditions

**Figure 2:**
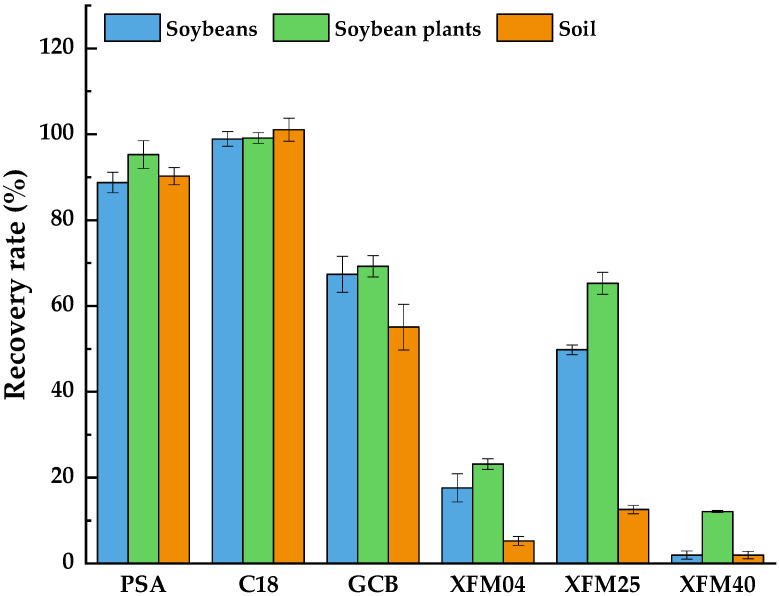
Effects of different purification agents on the recovery rate of pydiflumetofen added to soybeans, soybean plants, and soil.

In this study, the QuEChERS method was selected to clean the soybean, soybean plant and soil samples because of its common use and effectivity in sample purification. We compared the purification effect for the recovery rates of PSA, C_18_, GCB, and MWCNTs of different sizes at the exact dosage of 50 mg, selected because these are often used as adsorbents. The test results are shown in Figure 2. The recovery rate using MWCNTs and GCBs was less than 70%, which cannot satisfy the requirements of pesticide residue analysis. This may be due to the characteristics of MWCNTs and GCB, such as a large specific surface area and strong adsorption, as well as a strong adsorption effect on pydiflumetofen in the removal of impurities. When the purification agent was PSA, the recovery rates were 88.7–90.2%, the RSDs were 2.0–3.2%, and the MEs were 99.2–118.4%. For purification agent C_18_, the recovery rates were 98.9–101.1%, the RSDs were 1.2–2.7%, and the MEs were 100.7–106.1%. Thus, 50 mg C_18_ and 100 mg MgSO_4_ were selected as the purifiers for the soybean and soil matrices. Considering that plants contain a large number of potential interferents such as chlorophyll, MgSO_4_ and C_18_ have a weak ability to remove chlorophyll and GCB has a strong adsorption ability for the pigments in impurities. As 50 mg GCB has a strong adsorption capacity for pydiflumetofen, GCB was added at doses of 10, 15, and 20 mg to effectively remove impurities while obtaining a satisfactory recovery rate of 50 mg C_18_ and 100 mg MgSO_4_, comparing the purification effect on the soybean plant. The recovery rates were 104.2%, 97.9%, and 93.4%, respectively, and the ME was between 80% and 100%. When the GCB dose was 10 mg, the extract was light green. However, the extract was clear and transparent when the dose was increased to 15 and 20 mg. Ultimately, 15 mg GCB, 50 mg C_18_, and 100 mg MgSO_4_ were selected as the purification agents of the soybean plant matrix to reduce the cost and the environmental impact of the procedure.

### 2.4. Method Validation

#### 2.4.1. Linearity and ME

Soybean plant, soybean, and soil samples without pydiflumetofen were selected and treated according to the method described in Section 3.3. to obtain a blank matrix solution of three different samples. The pydiflumetofen standard was added to the blank matrix solution to formulate the matrix standard with concentrations of 0.001, 0.002, 0.005, 0.01, 0.02, 0.05, 0.1, 0.2, and 0.5 mg/L. Details of the chromatographic conditions are described in Section 3.4. The standard curve of the matrix standard was drawn with the pydiflumetofen concentration as the abscissa co-ordinate and the peak area as the ordinate co-ordinate. As shown in Table S1, the matrix-matched standard calibrations were used to calibrate possible interferences on the quantification of analytes as a compensatory strategy for Mes. The peak areas pydiflumetofen at 0.001–0.5 mg/kg in soybean plant, soybean, and soil are linearity related to the mass concentration, and the correlation coefficient® is higher than 0.997. Under the above chromatographic conditions, the LOD of pydiflumetofen in soybean plant, soybean, and soil ranged from 3.5 × 10^−4^–4.1 × 10^−3^, and the LOQ was 0.01 mg/kg (Table 1). MRM chromatograms, based on the quantitative ion and obtained for pydiflumetofen dissolved in either acetonitrile or in one of the three matrices, at 0.01 mg/kg concentration, are shown in Figure S4. The slope of the linear equation of the standard curve of the matrix standard curve was compared to that of the standard curve, and the ratio of the two was calculated to determine the sample ME, as shown in Table S1. The MEs of the soybean plant and soil were 94.2% and 91.5%, respectively, which was negligible, and the ME of soybean was 68.7%, which indicated that the matrix had a weakening effect. Therefore, in this study, the matrix-matched standard calibrations were used to calibrate the possible interferences in the quantification of analytes, as a compensatory strategy for MEs.

#### 2.4.2. Accuracy and Precision

A recovery experiment was performed to verify the accuracy and precision of the method. The concentrations of pydiflumetofen added to the soybean plant were 0.01, 0.05, 0.50, and 50.0 mg/kg, to soybeans were 0.01, 0.05, and 0.50 mg/kg, and to soil were 0.01, 0.05, 0.50, and 2.00 mg/kg. First, 1 mL standard solution with different concentrations of pydiflumetofen were added to the soybean plant, soybean, and soil in 50 mL centrifuge tubes and then left for 3 h to allow the solvent to evaporate. Next, they were treated according to the method described in Section 2.3, and the results are shown in Table 1. The average recovery rate of pydiflumetofen in soybean plant, soybean, and soil matrices ranged from 83.9% ± 1.1% to 99.5% ± 3.3%, and RSD (n = 5) from 0.77% to 7.77% (<10%).

The sensitivity, accuracy, and precision of the method chosen in this study met the requirements and were suitable for the residual detection and analysis of pydiflumetofen in soybean.

### 2.5. Degradation Dynamics of Pydiflumetofen in Soybean Plant and Soil

The degradation dynamics of pydiflumetofen were analyzed for 2 h–60 d after application, and the results are shown in Table 2. The initial deposits of pydiflumetofen in the plant and soil were 21.50–45.22 mg/kg and 0.49–1.16 mg/kg, respectively. The initial residues in the plant were higher than those in the soil samples, mainly because the soil samples were collected at a depth of 0–10 cm, and pydiflumetofen was presented on the surface of the soil, while the soil below acts to dilute the pydiflumetofen. The difference in the degradation rate of pydiflumetofen in the plant was low between the different test sites; however, the difference was high in the soil samples, presented as Hailun > Chifeng > Changchun. We speculated that the degradation rate of pydiflumetofen in the soil may be related to the environmental conditions at the experimental sites. As shown in Table S2, the soil organic matter of Hailun’s was 4.6%, Chifeng was 1.7%, and Changchun was 2.6%. The organic matter content in the Hailun soil was significantly higher than that in the soils of the other two locations, which could be the main reason why the digestion rate of pydiflumetofen in the Hailun soil was faster than the soil of the other two locations. The soil organic matter content in Changchun was slightly higher than that in Chifeng; however, the degradation rate was lower than that in Chifeng, which may be affected by factors such as the type of microorganisms in the soil, temperature, humidity of the environment, and the sunshine duration. 

**Figure 3:**
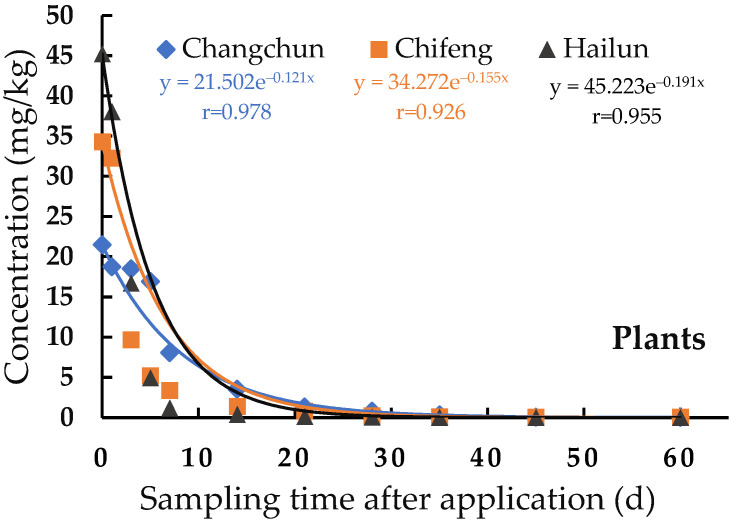

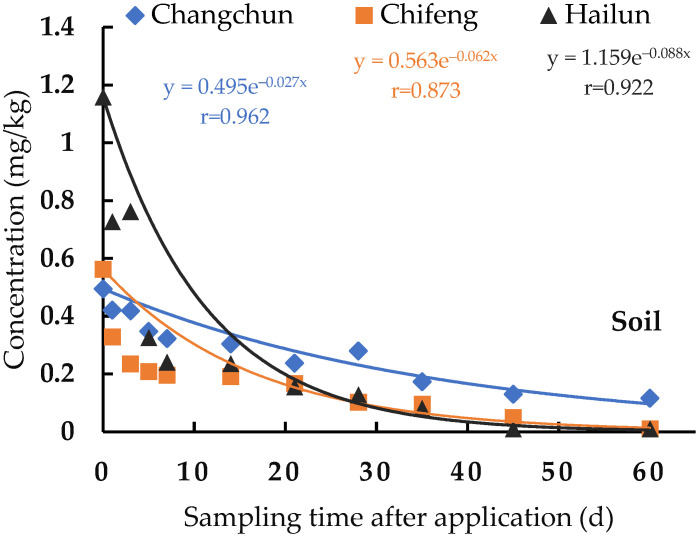
Dissipation of pydiflumetofen in plant and soil after application at the dosage of 225 mL a.i./ha.

The results demonstrated that the dissipation of pydiflumetofen in the plant and soil followed a first-order kinetic model (Figure 3 and Table 2). The residual amount was exponentially related to the number of days of application, and the half-life was 3.6–5.7 d and 7.9–25.7 d, respectively. The total half-life was less than 30 d, indicating that pydiflumetofen is an easily degradable pesticide.

Previous studies [13,14,16,23] have shown that the half-lives of pydiflumetofen in rice plant, wheat grains, watermelon, and bananas are 1.1–9.3 d, 3.2–4.4 d, 2.1–3.4 d, and 16.9 d, respectively, and the half-lives in paddy soil and watermelon soil are 6.08–14.38 d and 21–69.3 d, respectively, which are quite different from our experimental results. This indicates that different crops metabolize the pesticide differently. Different growth environments lead to different degradation rates of pydiflumetofen.

### 2.6. Terminal Residues of Pydiflumetofen in Soybean

Final residue detection in soybean is necessary for the safety evaluation of pesticides in soybean fields. Based on the chosen HPLC-MS/MS method, the final residual amount of pydiflumetofen in soybean was detected at the harvest stage at all three sites. The final residual concentrations of pydiflumetofen observed in soybean grown in Changchun, Chifeng, and Hailun ranged from less than 0.01 to 0.046 mg/kg (Table S3). China has not yet established an MRL value of pydiflumetofen in soybean. However, in this study, the maximum residue of pydiflumetofen detected in harvested soybean does not exceed the MRL value of pydiflumetofen in legumes which has been prescribed in other countries (Table S4). Therefore, the study recommends that the MRL value of pydiflumetofen in soybean in China is 0.06 mg/kg (calculated from OECD MRL Calculator Spreadsheet Single Data Set, https://www.oecd.org/env/ehs/pesticides-biocides/oecdmaximumresiduelimitcalculator.htm, accessed on 22 September 2022).

### 2.7. Dietary Risk Assessment

#### 2.7.1. Risk Assessment of Acute Diet

The HR value of pydiflumetofen in soybean was selected for acute dietary intake risk assessment, and the assessment results are shown in Table 3. According to the JMPR report, the ARfD of pydiflumetofen was 0.3 mg/kg bw, LP was 0.24 g/kg bw/day (obtained using the IESTI calculator, available at: https://zwfw.nhc.gov.cn/kzx/tzgg/tzggqb/, accessed on 22 September 2022), and bw was 63 kg, the average weight of adults in the Chinese population. The NESTI was calculated to be 0.01104 mg and the ratio of NESTI to ARfD was 0.06%.

#### 2.7.2. Risk Assessment of Chronic Diet

The pesticide information network shows that the registered crops of pydiflumetofen in China include wheat, potatoes, soybean, tomatoes, cucumbers, grapes, and peanuts. The MRL values of pydiflumetofen in various countries are listed in Table S4. The MRL values were in the following order: China > CAC > USA > EU > South Korea > Japan > Australia > Canada. The STMRi value of pydiflumetofen on soybean was selected for the long-term dietary intake risk assessment, and the assessment results are shown in Table 3. According to the JMPR report, the ADI of pydiflumetofen was 0.1 mg/kg bw. The ratio of NEDI to ADI was calculated to be 0.46 mg, and the ratio of NEDI to ADI was 7.54%.

The assessment results showed that the acute hazard index (aHI) value was 0.06%, and the risk quotient (RQ) value was 7.54%, which was less than 1, thereby indicating that it would not pose an unacceptable risk to public health.

## 3. Materials and Methods

### 3.1. Chemicals and Reagents

Chromatographic-grade methanol and acetonitrile were purchased from Thermo Fisher Scientific (Waltham, MA, USA), formic acid was purchased from Sigma–Aldrich (St. Louis, MO, USA), ammonium acetate and ethyl acetate were purchased from the Beijing Chemical Factory (Beijing, China), pydiflumetofen standard (purity 99.7%) was purchased from Dr. Ehrenstorfer GmbH (Augsburg, Germany), and 20% pydiflumetofen soluble concentrate (SC) was purchased from Syngenta Crop Protection Co., Ltd. (Kunshan, China).

Primary secondary amine (PSA, 40–60 μm), end-capped octadecyl carbon (C_18_, 50 μm), and graphitized carbon black (GCB, 38–125 μm) were purchased from Agela Technologies (Shanghai, China); Multiwalled carbon nanotubes (MWCNTs)–XFM40 (diameter 10–20 nm, length 5–30 μm), XFM25 (diameter 30–50 nm, length < 10 μm), and XFM04 (diameter 5–15 nm, length 0.5–2 μm) were purchased from Xianfeng Nanomaterials Technology Co., Ltd. (Nanjing, China). Wahaha Group Co., Ltd. (Hangzhou, China) supplied purified water. Sodium chloride (NaCl) and magnesium sulfate (MgSO_4_) were purchased from Guangdong Xilong Science Co., Ltd. (Shantou, China). Polyethylene centrifuge tubes of 5- and 50-mL volumes were purchased from Biosharp Corporation (Hefei, China), and an organic membrane of pore size 0.22 μm was purchased from Jinteng Experimental Equipment Co., Ltd. (Tianjin, China).

### 3.2. Field Trial

The experimental design followed the “Guidelines for Testing Crop Pesticide Residues”, edited by the Ministry of Agriculture and Rural Affairs of the People’s Republic of China [24]. In 2021, field experiments were conducted in three locations in China, and 20% pydiflumetofen SC was applied in the soybean fields at three sites: Changchun City, Jilin Province; Chifeng City, Inner Mongolia Autonomous Region; and Hailun City, Heilongjiang Province. The experimental sites consisted of treatment and control plots of 100 m^2^, which were sufficient to obtain a representative sample for each group, and each treatment included three replicates. The soil properties and climatic conditions at the three sites are listed in Table S2.

#### 3.2.1. Dissipation of Pydiflumetofen in Soybean Plant and Soil

To analyze the digestion dynamics, two treatments (1 and 2) were set up at each experimental site and were tested in triplicate. Treatment 1 was a soybean plant dynamics plot; treatment 2 was a soil dynamics plot. Pydiflumetofen SC at 20% concentration was applied once at a dose of 225 mL a.i./ha (1.5× highest recommended dose) to both plots. Samples were collected from 12 random points in each replicate plot at 2 h and at 1, 3, 5, 7, 14, 21, 28, 35, 45, and 60 d after application.

#### 3.2.2. Terminal Residue of Pydiflumetofen in Soybean

To analyze the final residue, four treatments (3, 4, 5, 6) and one control (no spraying of pydiflumetofen) were set up at each test point. In treatments 3 and 4, 150 mL a.i./ha (highest recommended dose) of 20% pydiflumetofen SC was administered three and four times, respectively, at an interval of 7 d, and this was repeated three times. In treatments 5 and 6, 225 mL a.i./ha (1.5 × highest recommended dose) 20% pydiflumetofen SC was administered three and four times, respectively, at an interval of 7 d, and this was repeated three times. Soybean samples were collected at 21, 28, and 35 d after the last application in each treatment group.

#### 3.2.3. Field Sample Preparation

Samples of soybean plants, soybeans with pods, and at least 2000 g of soil were collected from the field. Plant samples were chopped using a stainless-steel knife, mixed in a stainless-steel basin, and divided into three 300 g samples by the quartering method. Soybean samples were collected from soybean seeds after removing the pods, mixed thoroughly, and divided into three 300 g samples by the quartering method. Soil samples collected at depths of 0–10 cm were cleaned for impurities such as stone debris and dead plants, and three soil samples of 300 g each were obtained by the quartering method. All samples were placed in sealed sample bags, labeled, and stored at −20 °C until analysis. The sample preparation process is shown in Figure S5.

### 3.3. Sample Pre-Treatment

The soybean plants and soybean samples collected in the field were placed in a knife-type mixing grinder (GM 200, Retsch, Haan, Germany) and mashed for 5 min while the soil samples were sieved through a 1 cm sifter. Each sample from the terminal residue experiments was divided into two parts for direct analysis and processing (shown in Figure S5).

#### 3.3.1. Sample Extraction

Samples were extracted using the QuEChERS method. Samples of approximately 5.0 g (± 0.1 g) of soybean plant, soybean, and soil were weighed using a 1/100 balance (JJ500Y, Shimadzu Instrument Corporation, Kyoto, Japan) and added into a 50 mL centrifuge tube, followed by the addition of 5 mL of purified water and 15 mL of acetonitrile solution. The samples were homogenized for 1 min using a multifunctional vortex mixer (VORTEX-3, IKA Group, Staufen, Germany). Furthermore, 1 g of NaCl for salination and delamination and 4 g of MgSO_4_ for water adsorption were added into the centrifuge tube, shaken vigorously 100 times, and then centrifuged (TDL-5-A, Anke Instrument Factory, Shanghai, China) at 5000× *g* for 5 min. The supernatant (acetonitrile phase) was then collected.

#### 3.3.2. Sample Cleanup

The supernatant (1.5 mL) was placed in a 5 mL centrifuge tube containing the purifying agents. For the soybean and soil samples, MgSO_4_ (100 mg) and C_18_ (50 mg) were used as purifying agents, whereas MgSO_4_ (100 mg), C_18_ (50 mg), and GCB (10 mg) were used as purifying agents for plant samples. The mixture was shaken vigorously 100 times and centrifuged at 5000× *g* for 5 min. The supernatant was passed through a 0.22 μm membrane using a sterile syringe and analyzed by HPLC–MS/MS.

### 3.4. Instrumental Conditions

#### 3.4.1. Chromatographic Conditions

HPLC–MS/MS was performed using an Agilent 1260–6470 triple quadrupole mass spectrometer (Santa Clara, CA, USA) equipped with an Agilent C_18_ column (3.0 × 100 mm, 1.8 μm, Zorbax Rapid Resolution High-Definition Eclipse Plus). The HPLC conditions were as follows: mobile phase A; 0.1% formic acid + 5 mM ammonium acetate aqueous solution; mobile phase B acetonitrile solution. The gradient elution procedure was as follows: 0–2 min, 90–60% A; 2–3 min, 60–40% A; 3–5 min, 40–10% A; 5–7 min, 10% A; 7–9 min, 10–40% A; 9–9.5 min, 40–60% A; 9.5–10 min, 60–90% A; and 10–15 min, 90% A. The flow rate was 0.3 mL/min, the column temperature was 30 °C, and the injection volume was 5 μL.

#### 3.4.2. Mass Spectrometry Conditions

The ion source was electrospray ionization, and the operating conditions were as follows: capillary voltage, +4000 V, nebulizer, 45 psi; drying gas, nitrogen 11 L/min; dry gas temperature, 350 °C; sheath gas, nitrogen 12 L/min; sheath gas temperature, 350 °C. The optimized parameters for multiple reaction monitoring (MRM) mode were as follows: pydiflumetofen quantification ion value was 193.1 *m/z*, and those of qualitative ions were 406.1 and 193.1 *m/z*, at a fragmentor voltage of 110 V and collision energies of 13 eV (for the ion at *m/z* 406.1) and 43 eV (for the ion at *m/z* 193.1).

### 3.5. Preparation of Standard Solution

A 1/10,000 mass balance (AUY220 Shimadzu Instrument Corporation, Japan) was used to weigh 0.0100 g of pydiflumetofen which was transferred to a 10 mL volumetric flask, diluted with acetonitrile to a 1000 mg/L standard solution, and stored in a freezer at 4.0 °C with a shelf life of six months. The solution was diluted with acetonitrile to obtain a standard working solution of different concentrations (0.001, 0.002, 0.005, 0.01, 0.02, 0.05, 0.1, 0.2, and 0.5 mg/L). The corresponding amounts of blank solution, soybean plant, soybean, and soil filtrate were added to the standard working solution of known concentration to prepare a matrix standard solution.

### 3.6. Method Validation

The chosen analytical methods were validated in terms of accuracy, precision, sensitivity, and selectivity to ensure that the intended purpose was achieved. The accuracy of this method was assessed using the average recovery of the different added levels, and the relative standard deviation (RSD) was calculated as a measure of accuracy. To determine the intra-day accuracy, five replicates were performed for each sample. Inter-day accuracy measurement was repeated five times for each sample at different times and by different operators in the same experimental environment. To determine the sensitivity of the method, the limit of detection (LOD) of pydiflumetofen was calculated by the lowest concentration that produced a triple signal-to-noise ratio. The limit of quantification (LOQ) was defined as the lowest spiking level of pydiflumetofen for acceptable recovery [25]. The evaluation of the matrix effects (MEs) verified the selectivity and reliability of the proposed methods, and the MEs for each matrix were calculated using the following equation:

(2) $$\mathrm{ME}=\frac{\mathrm{Km}}{\mathrm{Ks}}\times 100\%$$

where Ks represents the standard curve slope of the acetonitrile standard solution, and Km represents the standard curve slope of the matrix standard solution. ME = 100% indicated there was no effect; 80% < ME < 120%, indicated a weak effect, which could be ignored; and ME < 80% or ME > 120% indicated a matrix enhancement or matrix inhibition effect, respectively.

### 3.7. Statistical Analyses

#### 3.7.1. Analysis of Dissipation Dynamics

The digestion dynamics of pydiflumetofen in soybean plant and soil were assessed according to the first-order kinetic equation and were calculated as follows:

(3) $$C_{t}={\;C}_{0}\times e^{-\mathrm{kt}}$$

(4) $$t_{1/2}=\frac{\ln2}{k}=\frac{0.693}{k}$$

where C_t_ is the residual concentration (mg/kg) of pydiflumetofen in the soybean plant or soil at time t; C_0_ is the initial concentration (mg/kg) of pydiflumetofen in the soybean plant or soil after application; k is the degradation rate constant; t is the time after the administration of pydiflumetofen (d); and t_1/2_ is the half-life of pydiflumetofen digestion.

#### 3.7.2. Dietary Risk Assessment

The measurement of the amount of final residue in soybean involved trials in three locations to assess the risk of acute dietary intake (aHI) of pydiflumetofen and chronic dietary intake risks (RQ). The aHI and RQ values for dietary exposure and risk assessment were estimated using the following equations:

(5) NESTI = HR×LP

(6) $$\mathrm{aHI}\;=\frac{\mathrm{NESTI}}{\mathrm{ARfD}\times \mathrm{bw}}$$

(7) $$\mathrm{NEDI}\;=\sum^{\;}\left(\mathrm{STMRi}\times \mathrm{Fi}\right)$$

(8) $$\mathrm{RQ}\;=\frac{\mathrm{NEDI}}{\mathrm{ADI}\times \mathrm{bw}}$$

where NESTI is the nationally estimated short-term intake; HR is the highest residual amount (mg/kg); LP is the residual amount for residents who consume large meals of soybean (kg/d); Fi is the average daily intake of soybean (kg/d); bw is the average weight of an adult in China (kg); ARfD is the acute reference dose (mg/kg bw); NEDI is the estimated daily intake nationwide; STMRi is the median residual amount (mg/kg) from supervised trials; and ADI is the acceptable daily intake (mg/kg bw).

## 4. Conclusions

Based on the QuEChERS multiresidue analysis method, a QuEChERS–HPLC–MS/MS detection method for pydiflumetofen in soybean was chosen. This method is simple, fast, sensitive, and accurate, and meets the requirements of rapid detection of pydiflumetofen in soybean. The method can be applied to the analysis of pesticide residues in field test samples for pesticide registration residues.

Under field conditions, after the application of 20% pydiflumetofen SC at 1.5 × the recommended dose, the half-lives of pydiflumetofen in soybean plant and soil were 3.6–5.7 d and 7.9–25.7 d, respectively. Pydiflumetofen is a readily degradable pesticide (t_1/2_ < 30 d).

After the application of 20% pydiflumetofen SC at 1.5 times the recommended dose, the residual amount of pydiflumetofen in soybean in the three locations was lower than the corresponding MRL in other countries. It is recommended that China establish an MRL value for pydiflumetofen in soybean of 0.06 mg/kg. The results of the dietary risk assessment of pydiflumetofen in soybean showed that aHI and RQ values of soybean harvested at maturity were below 1, indicating that at this dose there was no unacceptable risk to public health. This study provided data support for the rational use of a 20% pydiflumetofen SC on soybean in China and conducted a preliminary assessment of the safety of soybean consumption.

## Figures and Tables

**Table 1:** Intra- and inter-day average recovery (%, *n* = 5) with RSD, LODs, and LOQ of pydiflumetofen in soybean plant, soybean, and soil samples.

Matrices	Spiked Level (mg/kg)	Recovery (Mean ± SD)		RSD		LOD mg/kg	LOQ mg/kg
		Intra-Day	Inter-Day	Intra-Day	Inter-Day		
soybean plant	0.01	92.9 ± 3.2	90.8 ± 4.0	3.48	4.43	2.4 × 10^−3^	0.01
	0.10	94.1 ± 2.2	83.9 ± 1.1	2.36	1.31		
	0.50	98.7 ± 2. 9	88.7 ± 2.0	2.93	2.23		
	50.0	97.6 ± 0.8	97.9 ± 0.7	0.81	0.77	4.1 × 10^−3^	
soybean	0.01	99.5 ± 3.3	96.1 ± 6.5	3.27	6.79		
	0.10	97.9 ± 0.9	93.6 ± 7.3	0.89	7.77		
	0.50	98.7 ± 0.89	91.8 ± 1.9	0.81	2.30		
soil	0.01	99.4 ± 1.19	96.6 ± 5.1	1.10	5.30	3.5 × 10^−4^	
	0.10	93.0 ± 3.09	93.6 ± 3.0	3.22	3.00		
	0.50	94.8 ± 2.7	93.3 ± 2.6	2.81	2.78		
	2.00	96.0 ± 3.2	94.3 ± 2.9	3.30	3.07		

Note: RSD, relative standard deviation; LOD, limit of detection; LOQ, limit of quantification; SD, standard deviation.

**Table 2:** Degradation dynamics equation, correlation coefficient and half-life of pydiflumetofen in plant and soil.

Matrices	Location	Equation	Coefficient (r)	Half-Life (t_1/2_)
Soybean plant	Changchun	C_t_ = 21.502 × 10^−0.121t^	0.978	5.7
	Chifeng	C_t_ = 34.272 × 10^−0.155t^	0.926	4.5
	Hailun	C_t_ = 45.223 × 10^−0.191t^	0.955	3.6
Soil	Changchun	C_t_ = 0.495 × 10^−0.027t^	0.962	25.7
	Chifeng	C_t_ = 0.563 × 10^−0.062t^	0.873	11.2
	Hailun	C_t_ = 1.159 × 10^−0.088t^	0.922	7.9

Note: t, time.

**Table 3:** Risk assessment of short- and long-term dietary intake of pydiflumetofen in soybean based on the Chinese dietary pattern.

Food Category	Fi(kg/d) ^a^	Commodity	MRLs ^b^	STMRi ^b^	HR ^b^	Source of Reference Limit
Wheat cereals and wheat products	0.1385	Wheat	0.6			Japan
Potatoes	0.0495	Potato	0.5			CAC ^c^
Dried beans and their products	0.016	Soybean		<0.010	0.046	PHI ^d^ of 21 days
Dark-colored vegetables	0.0915	Tomatoes	2.0			South Korea
Light-colored vegetables	0.1837	Cucumber	0.5			USA
Fruits	0.0457	Grape	2.0			Japan
Oilseeds and oil	0.0327	Peanut	0.02			USA
Total NEDI (mg)	0.4749					
NESTI (mg)	0.0110					
ADI (mg/kg bw)	0.1					
ARfD (mg/kg bw)	0.3					
LP (g/kg bw/day)	0.24					
Body weight (kg bw)	63					
aHI (%)	0.06%					
RQ (%)	7.54%					

^a^ Consumption values of soybean and other crops refer to the recommended dietary food intake (Fi) of an adult (63 kg) per day for its corresponding food classification (data from the dietary guidelines published by the Health Ministry of the People’s Republic of China). ^b^ Supervised trial median residue (STMRi) in soybean and maximum residue limits (MRLs) in other crops were used to calculate the national estimated daily intake (NEDI). The high residue (HR) in soybean was used to calculate the national estimated short-term intake (NESTI). ^c^ CAC, Codex Alimentarius Commission. ^d^ PHI, Pre-harvest interval.

## Data Availability

Not applicable.

## Supplementary Materials

The following supporting information can be downloaded at: https://www.mdpi.com/article/10.3390/molecules27238465/s1, Figure S1: Chemical structure of pydiflumetofen. Figure S2: 1.0 mg/L chromatograms of pydiflumetofen. A: The TIC chromatograms in different mobile phases; B: Quantitative ion MRM chromatograms of pydiflumetofen; C: Qualitative ion MRM chromatogram of pydiflumetofen; D: Spectrum of two product ions with *m*/*z* values of 193.1 and 406.1; Figure S3: Extraction of soybean and soil compared with and without the addition of water in acetonitrile and methanol. Figure S4: MRM chromatogram of pydiflumetofen at 0.01 mg/kg based on the quantitative ion. A: Pydiflumetofen dissolved in acetonitrile; B: Pydiflumetofen dissolved in plant matrix; C: Pydiflumetofen dissolved in soybean matrix; D: Pydiflumetofen dissolved in soil matrix. Figure S5: Sample preparation process. A: Collection and preparation; B: Extraction and purification. Table S1: Calibration equation, correlation coefficients (r), and matrix effects of pydiflumetofen in soybean plant, soybean and soil matrices. Table S2: Soil properties and climatic conditions at the experimental locations in China. Table S3: Terminal residues of pydiflumetofen in soybean with different application dosages and application times (n = 3). Table S4: MRLs adopted by other countries or organisms for pydiflumetofen in crops/products for which it is registered in China.

## Sample Availability

Samples of the compounds are not available from the authors.
