# Supplementary material for: Dissipation and Dietary Risk Assessment of Pydiflumetofen Residues in Soybean
Source: Molecules. 2022 Dec 2;27(23):8465. doi: 10.3390/molecules27238465 (PMC9738132; doi:10.3390/molecules27238465)
Supplement: Supplementary file 1 [file molecules-27-08465-s001.zip › molecules-1977042-supplementary.pdf]

Supplementary materials for

Dissipation and Dietary Risk Assessment of Pydiflumetofen Residues in Soybean

Liping Wei[1], Xingang Hou[2], Zhiguang Hou[1,*] Xiaolong Yu[1], Xiumei Wang[1], Qinghui Zhao[1], Hemin Gao[1], Hanju Liu[1], Xiaodong Zheng[1], and Zhongbin Lu[1],

1 College of soy Protection, Jilin Agricultural University, Changchun 130118, China

2 College of Science, China Agricultural University, Beijing 100193, China

* Corresponding author: E-mail: zhiguangh@jlau.edu.cn; Tel: +86 (431) 84510942; Fax: +86 (431) 84510942.

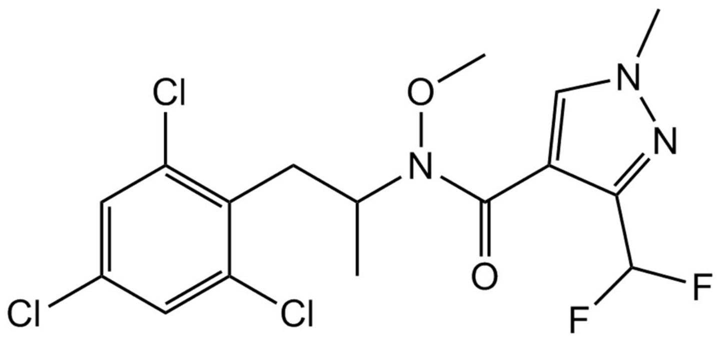

Figure S1. Chemical structure of pydiflumetofen

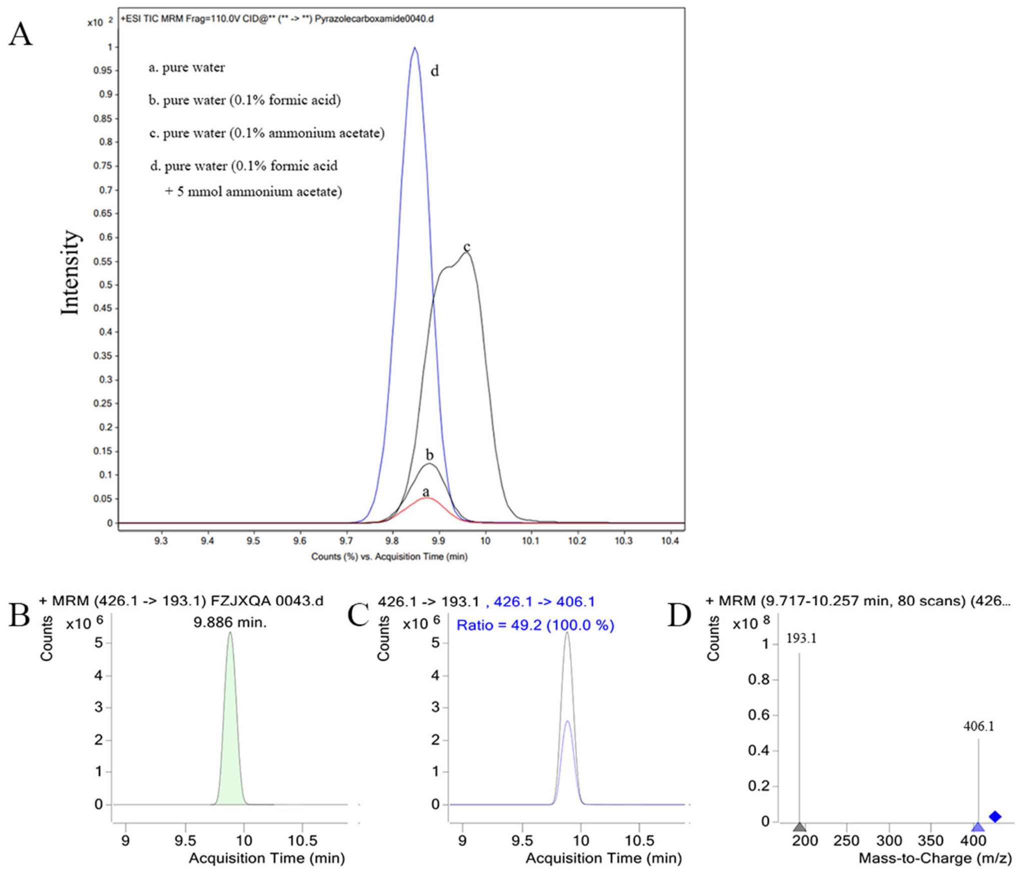

Figure S2. 1.0 mg/L chromatograms of pydiflumetofen. (**A**) The TIC chromatogram in different mobile phases; (**B**) Quantitative ion MRM chromatograms of pydiflumetofen; (**C**) Qualitative ion MRM chromatogram of pydiflumetofen; (**D**) Spectrum of two product ions with *m*/*z* of 193.1 and 406.1

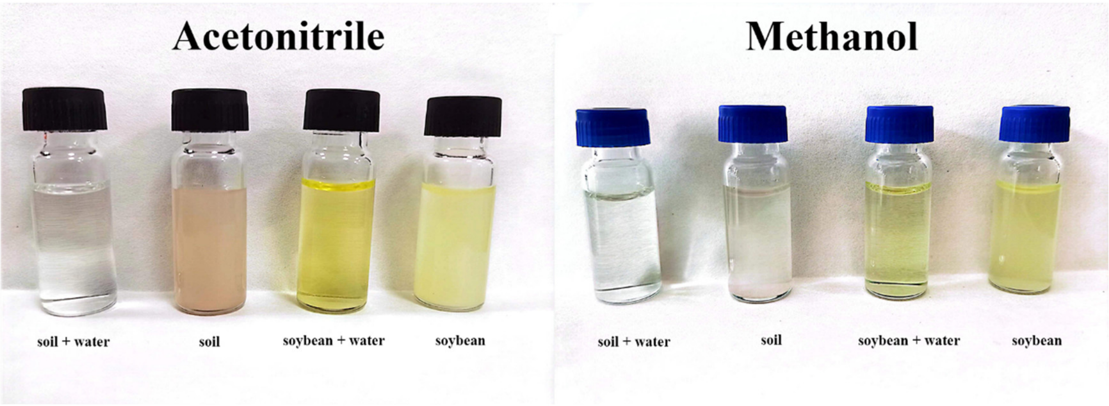

Figure S3. Extraction of soybean and soil compared with and without the addition of water in acetonitrile and methanol

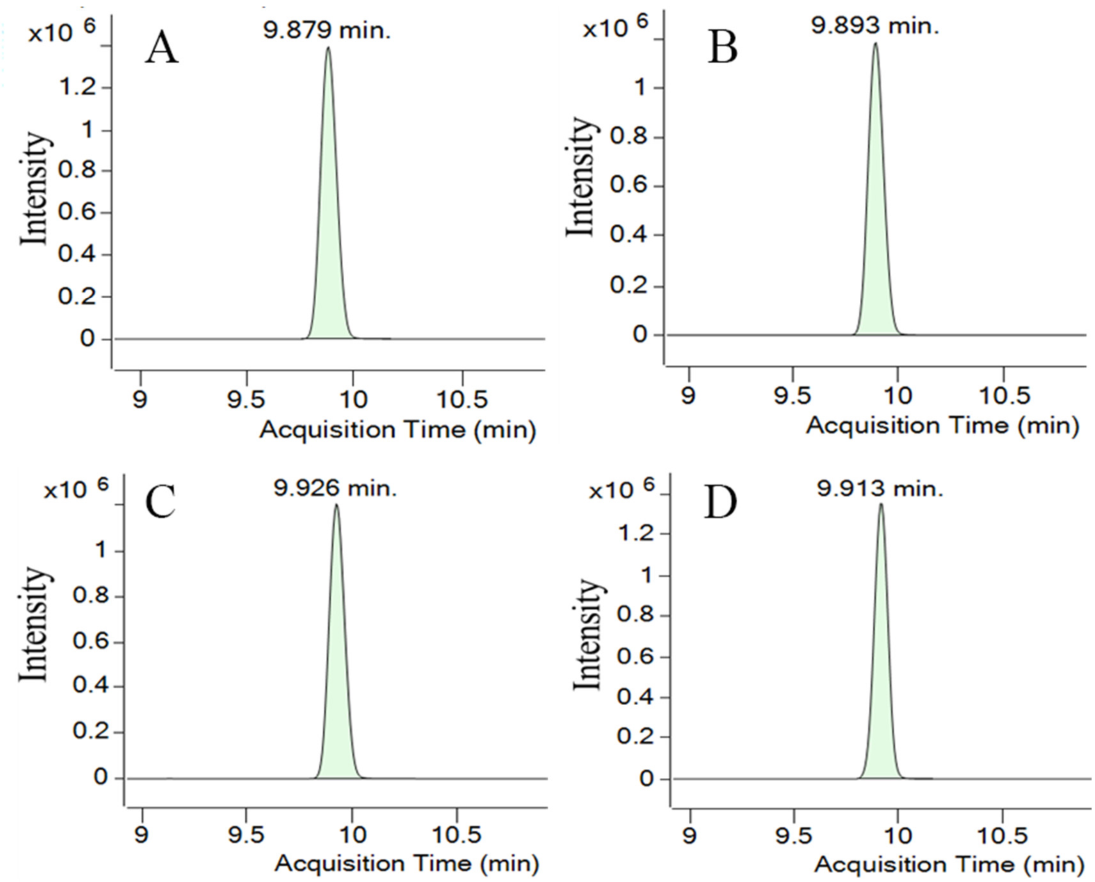

Figure S4. MRM Chromatogram of pydiflumetofen at 0.01 mg/kg based on the quantitative ion. (**A**) Pydiflumetofen dissolved in acetonitrile; (**B**) Pydiflumetofen dissolved in plant matrix; (**C**) Pydiflumetofen dissolved in soybean matrix; (**D**) Pydiflumetofen dissolved in soil matrix.

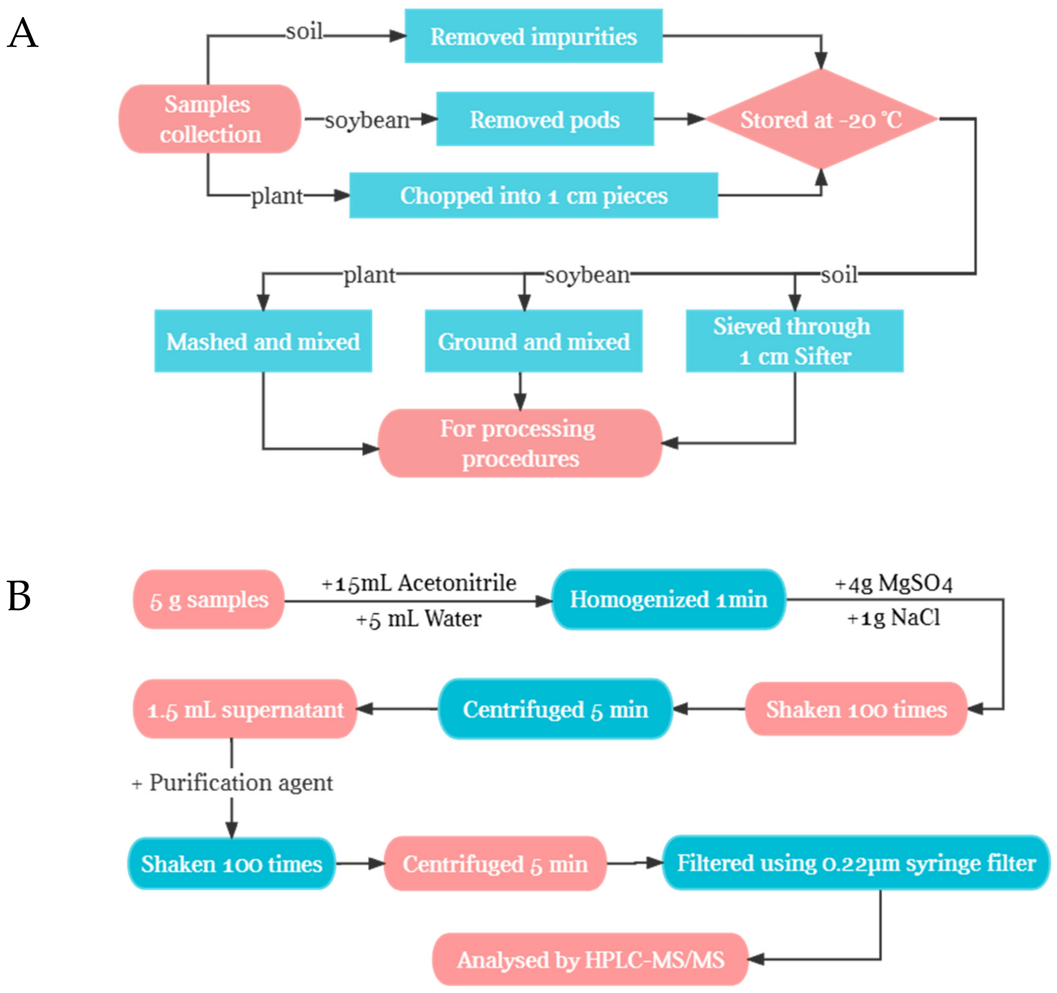

Figure S5. Samples preparation process. (**A**) Collection and preparation; (**B**) Extraction and purification.

Table S1. Calibration equation, correlation coefficients (r) and matrix effects of pydiflumetofen in soybean plant, soybean and soil matrices

Matrices	Range (mg/kg)	Matrix effects (%)	Calibration equation	r
Soybean plant	0.001~0.5	94.2	y=57,897,625.74x+317,536.33	0.9989
Soybean	0.001~0.5	68.7	y=26,725,890.67x+43,653.01	0.9999
Soil	0.001~0.5	91.5	y=35,576,480.23x+265,585.33	0.9992

Table S2. Soil properties and climatic conditions at the experiment location in China

Location	Soil classification	Organic matter (%)	pH	Mean Sunshine (h)	Total rainfall (Jun–Oct，mm)	Mean temperature (◦C)
Changchun	Meadow soil	2.6	6.9	2827.0	1408	18.7
Chifeng	Phaeozems	1.7	8.4	2681.8	501	18.1
Hailun	Phaeozems	4.6	5.3	2543.2	1129	16.6

Table S3. Terminal residues of pydiflumetofen in soybean with different application dosages and different application times (n = 3)

Dosage (g a.i./ha)	Times	PHI [a]	Residues data (n = 3) (Mean value, mg/kg)		
			Changchun	Chifeng	Hailun
150	3	21	<0.010	0.046	<0.010
		28	<0.010	0.034	<0.010
		35	<0.010	0.017	<0.010
150	4	21	<0.010	0.027	<0.010
		28	<0.010	0.010	<0.010
		35	<0.010	0.021	<0.010
225	3	21	<0.010	0.038	<0.010
		28	<0.010	0.014	<0.010
		35	<0.010	0.015	<0.010
225	4	21	<0.010	0.043	<0.010
		28	<0.010	0.015	<0.010
		35	<0.010	0.024	<0.010

Note: [a] PHI, Pre-harvest interval

Table S4. MRLs adopted by other countries or organisms for pydiflumetofen in crops/products for which it is registered in China

Food classification	China	CAC[a]	USA	EU	South Korea	Japan	Australia	Canada
Wheat cereals and wheat products		0.4	0.3		0.3	0.6		
Potatoes		0.5			0.015	0.02		
Dried beans and their products		0.4	0.4		0.4	0.4	0.5*	0.1
Dark-colored vegetables		15	50		40	40	30*	50
Light-colored vegetables		15	50		30	40	30*	50
Fruits		4	5		5	2	5*	5
Oilseeds and oil		0.9	0.9		0.9	0.9	0.07*	30

Note: [a] CAC: Codex Alimentarius Commission
